# Cryptic Disseminated Tuberculosis: a Secondary Analysis of Previous Hospital-Based Study

**Published:** 2020-01

**Authors:** Fahmi Yousef Khan

**Affiliations:** Department of Medicine, Hamad General Hospital, Doha-Qatar

**Keywords:** Cryptic tuberculosis, Disseminated tuberculosis, Sputum culture, Miliary tuberculosis

## Abstract

**Background:**

The main purpose of this study was to describe the demographic and clinical features of cryptic disseminated TB; it was also aimed to shed light on diagnostic test, procedure results, organ involvement, and outcomes of cryptic disseminated TB in patients with confirmed disseminated TB.

**Materials and Methods:**

We performed a secondary post hoc analysis of collected data from our previous study entitled “Disseminated Tuberculosis among Adult Patients Admitted to Hamad General Hospital, Qatar: A Five-Year Hospital-Based Study” with modified objectives. This study included patients admitted from January 1, 2006 to December 31, 2010.

**Results:**

Twenty-three patients were recruited with non-miliary patterns on chest x-ray. Their mean age was 34.4±12.6 years and 15 (65.6%) were males. The mean duration of illness was 46.13±48.4 days and the most common presenting symptom was fever in 20 patients (87%), while 3 (13%) patients had underlying medical conditions with diabetes mellitus 2 (8.7%), being the most common. Bronchoalveolar lavage (BAL) and bronchial wash (BW) fluids were Acid-fast bacilli (AFB) positive in 1/4 (25%) of the cases and culture-positive for *Mycobacterium tuberculosis* (*M. tuberculosis*) in 4/4 (100%) of all the cases. Two patients (8.7%) had positive sputum smear, while 18 (78.3%) patients had positive culture for *M. tuberculosis*. All except one patient completed their treatment in Qatar. One patient died one month after the start of antituberculous treatment.

**Conclusion:**

Cryptic disseminated TB should be suspected when a patient from TB-endemic countries develops unexplained fever and cough despite normal or non-miliary pattern chest radiograph. Moreover, respiratory specimen cultures should be obtained from these patients, regardless of the symptoms presented and the initial site of the involved organ.

## INTRODUCTION

Disseminated tuberculosis (TB) is defined as mycobacterial disease that has two or more non-contiguous sites resulting from hematogenous dissemination of *Mycobacterium tuberculosis* (*M.tuberculosis*). It may result from progressive primary infection or occur via reactivation of a latent focus with subsequent spread or rarely through iatrogenic origin (1,2). The term cryptic disseminated TB describes patients who have disseminated TB with less “typical” chest radiographic abnormalities, including normal and non-miliary pattern (3–6). It has an insidious form of presentation that mainly affects the middle-aged and elderly (3,4). Diagnosing disseminated TB in such patients is still a dilemma, from both a clinical and laboratory perspective because of the lack of localizing signs, absence of choroidal tubercles, normal chest x-rays, and negative tuberculin skin test. Currently available data on this clinical entity worldwide are sparse and of limited quality (3–6); this prompted us to perform this post hoc analysis. The purpose of this study was to describe the demographic and clinical features, diagnostic tests, procedural results, and outcomes of cryptic TB in patients with disseminated TB.

## MATERIALS AND METHODS

### Design and Setting

We performed a secondary post hoc analysis of collected data from our previous study entitled “Disseminated Tuberculosis among Adult Patients Admitted to Hamad General Hospital, Qatar: A Five-Year Hospital-Based Study” (1) with modified objectives as mentioned above. The primary outcome was also modified as, with the help of different diagnostic modalities, most of the patients were diagnosed with cryptic TB. The primary retrospective observational study was conducted at Hamad General Hospital, which is a tertiary center covering all specialties except for cardiology, hematology-oncology, and obstetrics. It involved all patients 15 years of age or older who were admitted to Hamad general hospital with disseminated TB from January 1, 2006 to December 31, 2010.

### Case Definition and Criteria for Diagnosis

Cryptic disseminated TB is a form of disseminated TB with non-miliary pattern or normal chest x-rays. It was diagnosed when the patient had normal or non-miliary pattern chest radiographs plus one of the following conditions:

1. Positive culture for *M. tuberculosis* from bone marrow, from liver biopsy specimen, or in two or more non-contiguous organs
2. Positive culture for *M. tuberculosis* from one organ and histopathological demonstration of caseating granulomas from another non-contiguous organ

Patients with miliary pattern chest x-rays were excluded from this study.

### Ethical Consideration

Since this was a secondary post hoc analysis of collected data from our previous study, no ethics committee approval or informed consent was required. The original study was approved by Medical Research Ethical Committee at Hamad Medical Corporation, Qatar (approval no. 12080/12).

### Data Analysis

The SPSS software (v 17.0; IBM Corp, Armonk, NY, USA) was used for data analysis and post-analysis results of continuous variables were expressed as means and standard deviations (SD).

## RESULTS

Of the 100 patients with disseminated TB in the primary study, we found 23 patients with cryptic disseminated TB accounting for 23% of all cases. Their mean age was 34.4±12.6 (range 21–67 days) and 15 (65.6%) patients were males, while 8 (34.4%) were females (Table 1).

The mean duration of symptoms prior to presentation was 46.13±48.4 (range 5–180 days) and the most common presenting symptoms were fever in 20 patients (87%), cough in 15 (65.2%), and anorexia in 15 (65.2%). Of all, 3 (13%) patients had underlying medical conditions with diabetes mellitus 2 (8.7%) being the most common, while no patient had human immunodeficiency virus (HIV) infection (Table 1). There was no history of contact with tuberculosis cases in 4.3% (1/23) of the patients, while only one patient’s family member had history of tuberculosis. No patient had a prior history of tuberculosis.

**Table 1.** Clinical characteristics of patients involved in this study

**Variable**	**Mean±SD(range)/(N%)**
**Age** [mean±SD(range: years)]	34.4±12.6(21–67)
**Age group**	
15–24	4(17.4%)
25–34	12(52.2%)
35–44	3(13%)
45	4(17.4%)
**Sex**	
M	15(65.6%)
F	8(34.4%)
**Nationality**	
Nepalese	9(39.1%)
Indian	3(13%)
Qatari	3(13%)
Ethiopian	2(8.7%)
Indonesian	2(8.7%)
Bangladeshi	1(4.3%)
Kyrgyzstan	1(4.3%)
Sri Lankan	1(4.3%)
Filipino	1(4.3%)
**Clinical presentation**	
Fever	20(87.0%)
Cough	15(65.2%)
Anorexia	15(65.2%)
Night sweat	14(60.9%)
Chills	12(52.2%)
Weight loss	11(47.8%)
Vomiting	11(47.8%)
Weakness	9(39.1%)
Headache	9(39.1%)
Abdominal pain	9(39.1%)
Chest pain	4(17.4%)
Dyspnea	3(13.0%)
Scrotal swelling	2(8.7%)
Hemoptysis	2(8.7%)
Pleural effusion	8(34.8%)
Enlarged lymph nodes	7(30.4%)
Ascites	6(26.1%)
Splenomegaly	4(17.4%)
Hepatomegaly	4(17.4%)
Meningitis	3(13.0%)
Others	9(39.1%)
**Associated medical conditions**	
Diabetes mellitus	2(8.7%)
ESRD	1(4.3%)
Duration of illness (days)	46.13±48.4(5–180)
Duration of treatment (months)	7.2±2.2(1–10)
**Outcome**	
Alive	22(95.7%)
Died	1(4.3%)

Tuberculin skin tests were positive in 43.5% (10/23) of the cases and erythrocyte sedimentation rate ranged between 2 and 137 mm/h (mean 49.42±30.60). Table 2 summarizes the main hematological findings in our patients.

**Table 2.** Hematological findings for patients involved in this study

**Variable**	**Mean ± SD (range)/N (%)**
**WBC/μL**	9980±44790 (4400–23600)
**Hemoglobin (gm/dL)**	11.3±2.2 (7–15)
**Platelets/μL**	319000±130000 (160000–635000)
**ESR (mm/hour)**	50.2±27.2 (9–104)

The chest radiograph and CT scan chest were normal in 3(13%) cases and abnormal but nonmiliary in 20(87%) cases. Chest radiography findings with nonmiliary pattern include consolidation 7(30.4%), cavities 4(17.4%), calcification 1 (4.4%), and pleural effusion 8(34.8%).

Bronchoalveolar lavage (BAL) and Bronchial wash (BW) fluids were Acid-fast bacilli (AFB) positive in 1/4(25%) of the cases and culture-positive for *M. tuberculosis* in 4/4 (100%) of the cases, while two patients (8.7%) had positive sputum smear and 18 (78.3%) patients had positive culture for *M. tuberculosis*. Table 3 describes the histopathological and microbiological characteristics of different specimens. The lung was the most frequently involved organ (Table 4).

**Table 3.** Histopathological and microbiological results for different spacemen from disseminated tuberculosis patients involved in this study

**Spacemen**	**Caseating granuloma**	**AFB smear positive (%)**	**TB culture positive (%)**
**Sputum**	NA	2/23 (8.7%)	18/23 (78.3%)
**CSF**	NA	0/5	4/5 (80%)
**BAL**	NA	1/4 (25%)	4/4 (100%)
**BW**	NA	1/4 (25%)	4/4 (100%)
**Ascitic fluid**	NA	0/5	5/5 (100%)
**Prostate aspirate**	NA	1/1 (100%)	1/1 (100%)
**Synovial fluid**	NA	1/2 (50%)	2/2 (100%)
**Bone/Bone marrow**	5/6 (83.3%)	1/6 (16.7%)	3/6 (50%)
**Lymph node biopsy**	2/2 (100%)	ND	ND
**Lymph node FNA**	6/6 (100%)	5/6 (83.3%)	5/6 (83.3%)
**Peritoneal biopsy**	1/1 (100%)	0/1	0/1
**Gastric biopsy**	1/1 (100%)	ND	ND
**Intestinal biopsy**	1/2 (50%)	2/2 (100%)	2/2 (100%)

CSF: cerebrospinal fluid; BAL: Bronchoalveolar lavage; BW: bronchial wash; NA: not applicable; FNA: fine needle aspiration; ND: not done

**Table 4.** The frequency of involved organs in disseminated tuberculosis patients presented at Hamad general hospital, Qatar during 2006 to 2010.

**Organ**	**N (%)**
**Lung**	20 (87%)
**Lymph nodes**	8 (34.8%)
**Pleura**	7 (30.4%)
**Peritoneum**	6 (26.1%)
**Meninges**	6 (26.1%)
**Bone**	4 (17.4%)
**Testis & Epididymis**	3 (13%)
**Intestine**	2 (8.7%)
**Joint**	1 (4.3%)
**Bone marrow**	1 (4.3%)

Treatment was initiated for all patients; initially, they received a combination of isoniazid, rifampicin, pyrazinamide, and either ethambutol or streptomycin for two months followed by isoniazid and rifampicin for 4 to 8 months. The mean duration of treatment was 7.2±2.2 (range 1–10 months). All except one patient completed their treatment in Qatar. One patient died one month after the start of antituberculous treatment.

## DISCUSSION

Disseminated TB can manifest with a variable clinical picture that resembles many other diseases, making the diagnosis on initial presentation difficult, and patients usually proceed undiagnosed until autopsy especially in the absence of typical miliary infiltrates on chest x-ray (3,4, 7). It was found in 0.3 to 13.3% of all autopsies (7).

Although cryptic disseminated TB is a well-known clinical entity, reports concerning this infection worldwide are sparse and of limited quality. To our knowledge, this is one of the few reports describing this disease in patients during their lifetime.

In their reports on cryptic miliary tuberculosis, Vasankari et al. and Yu et al. (3,4) found that the most affected age group were elderly patients and mostly females. Long et al. (8) also described female predominance with mean age of 48 years in patients with non-miliary pattern disseminated TB. On the contrary, our study showed a male predominance with a mean age of less than 34 years. The reason for this difference is unclear; however, it may reflect a change in the epidemiology of this disease. On the other hand and in agreement with other reports (3,4, 8), we found that all patients in our series were from TB-endemic countries and most of them presented with prolonged fever, cough, and anorexia (mean duration of symptoms prior to presentation was 46 days). Therefore, the diagnosis of cryptic disseminated TB requires a high index of suspicion. It should be suspected in patients from TB-endemic countries, who presented with unexplained fever, anorexia, and cough, despite the absence of miliary pattern on chest radiography.

It was suggested that the presence of underlying diseases such as HIV infection, chronic renal disease, diabetes, immunosuppression, and endocrine disorder may alter the typical presentation, which often delay or lead to missed diagnosis of cryptic tuberculosis (3,6). In our study, no patient had HIV infection and only 3 (13%) patients had underlying disease, suggesting that the role of underlying diseases in delaying or missing the diagnosis was not significant.

There is no consensus on the diagnostic workup of cryptic disseminated TB; however, the aim of any workup is to identify the involved site in order to obtain appropriate specimens to send for AFB smear, polymerase chain reaction (PCR), mycobacterial culture, and histology test (9,10). In the present study, in addition to the chest x-ray, various diagnostic approaches had been applied. None of them showed superiority over others. Instead, combinations of these diagnostic modalities were used to help in establishing the diagnosis of disseminated TB in most of our patients.

In our study, chest x-rays and CT scan chest were normal in 3 patients and disseminated TB was confirmed in this patients from other samples. In the remaining 20 cases chest radiography was abnormal but of nonmiliary pattern. The preliminary investigations such as sputum for AFB were negative in 18 (78.3%). Disseminated TB was confirmed from other samples. Interestingly, we found that BAL and BW fluids were culture-positive for mycobacterial TB in 4/4 (100%) of the cases and sputum cultures were positive in 78.3% of patients (18/23), generally highlighting the importance of obtaining cultures of respiratory samples in the evaluation of people with suspected cryptic disseminated TB. The high diagnostic yield of respiratory samples could be explained by the fact that the lung is the most common primary site from which TB infection could get into blood and disseminate to other organs (11). Therefore, respiratory specimens should be obtained for AFB, PCR, and mycobacterial culture from all patients with suspected disseminated TB, regardless of the symptoms presented and the initial site involved.

As noted in this study, the mortality was 4.3% which is less than the range of 46–80% reported in the literature (6,8).

There were limitations to this study. First, it was retrospective with a small sample size. Second, it was hospital-based. Third, PCR study on different specimens, especially respiratory, was not used because it was not available in our laboratory during the study period. Despite this, we believe that this study is the first step in exploring the details of this clinical entity in Qatar. Therefore, we recommend conducting large prospective studies to confirm our findings.

In conclusion, cryptic disseminated TB is a recognised clinical entity which poses a significant diagnostic challenge for clinicians. It should be suspected when a patient from TB-endemic countries has unexplained fever and cough despite a normal or non-miliary pattern chest radiography. Moreover, respiratory specimen cultures should be obtained from these patients, regardless of the symptoms presented and the initial site of the involved organ.

## References

[1] Khan FY Dosa K Fuad A Ibrahim W Alaini A Osman L Disseminated tuberculosis among adult patients admitted to Hamad general hospital, Qatar: A five year hospital based study. Mycobact Dis 2016;6(212):2161–1068.

[2] Salem B. Disseminated tuberculosis following the placement of ureteral stents: a case repot. Cases J 2008;1(1):383. 19077197 10.1186/1757-1626-1-383 PMC2614942

[3] Vasankari T Liippo K Tala E. Overt and cryptic miliary tuberculosis misdiagnosed until autopsy. Scand J Infect Dis 2003;35(11–12):794–6. 14723351 10.1080/00365540310016961

[4] Yu YL Chow WH Humphries MJ Wong RW Gabriel M. Cryptic miliary tuberculosis. Q J Med 1986;59(228):421–8. 3749446

[5] Ozaras R Vatankulu B Mete B Kacmaz B Halac M. Cryptic miliary tuberculosis. QJM 2016;109(10):689–690. 27435669 10.1093/qjmed/hcw111

[6] Patel D Dosi R. Cryptic tuberculosis: a missed diagnosis and an unusual presentation. Int J Res Med Sci 2016;4:5463–5.

[7] Savic I Trifunovic-Skodric V Mitrovic D. Clinically unrecognized miliary tuberculosis: an autopsy study. Ann Saudi Med 2016;36(1):42–50. 26922687 10.5144/0256-4947.2016.42 PMC6074281

[8] Long R O’Connor R Palayew M Hershfield E Manfreda J. Disseminated tuberculosis with and without a miliary pattern on chest radiograph: a clinical-pathologic-radiologic correlation. Int J Tuberc Lung Dis 1997;1(1):52–8. 9441059

[9] Sharma SK Mohan A Sharma A. Miliary tuberculosis: A new look at an old foe. J Clin Tuberc Other Mycobact Dis 2016;3:13–27. 31723681 10.1016/j.jctube.2016.03.003 PMC6850233

[10] Wang JY Hsueh PR Wang SK Jan IS Lee LN Liaw YS Disseminated tuberculosis: a 10-year experience in a medical center. Medicine (Baltimore) 2007;86(1):39–46. 17220754 10.1097/MD.0b013e318030b605

[11] Smith I. Mycobacterium tuberculosis pathogenesis and molecular determinants of virulence. Clin Microbiol Rev 2003;16(3):463–96. 12857778 10.1128/CMR.16.3.463-496.2003 PMC164219

